# Support model for nurses caring for people living with HIV and AIDS in the Limpopo province, South Africa

**DOI:** 10.4102/curationis.v44i1.2092

**Published:** 2021-07-06

**Authors:** Dorah U. Ramathuba, Lufuno Makhado

**Affiliations:** 1 Department of Advanced Nursing Science, Faculty of Health Sciences, University of Venda, Thohoyandou, South Africa; 2 Department of Public Health, Faculty of Health Sciences, University of Venda, Thohoyandou, South Africa

**Keywords:** caring, HIV and AIDS, job satisfaction, nurses, organisational support

## Abstract

**Background:**

Human immunodeficiency virus (HIV) and acquired immune deficiency syndrome (AIDS) caregiving has created a foundation for stress and burnout amongst nurses as they are burdened by the increased workload of HIV and AIDS care.

**Objectives:**

This study aimed to develop a support model for nurses caring for people living with HIV and AIDS (PLWH).

**Method:**

The study employed concept analysis and the identified concept of interest within the caring context of HIV and AIDS was conceptualised using six elements of practice-oriented theory, namely, the context, agent, recipient, dynamic, procedure and purpose.

**Results:**

The framework consisted of six components: health service and legislative (context); nursing service managers (agents); nurses (recipients); decreasing power imbalance, participative and transformational leadership and trust (dynamics); initiation of support process through teamwork and mutual goal-setting, implementation and sustenance through reflections, monitoring and evaluation (process) and empowered nurses (outcome).

**Conclusion:**

Poor organisational support deteriorates the quality of nurses’ lives on a personal level and imposes a direct economic cost on the employer by decreasing overall nursing workforce productivity. The conceptual framework could be a guide to support nurses in healthcare services regarding the management of HIV and AIDS in the workplace.

## Introduction

The overall prevalence rate of human immunodeficiency virus (HIV) in South Africa was 11.2% and the total number of people living with HIV (PLWH) was estimated to be approximately 6.19 million in 2015 (Statistics South Africa [StatsSA] 2015). Human immunodeficiency virus and acquired immune deficiency syndrome (AIDS) are on a vertical upsurge, with about 100 000 additional PLWH yearly (Makhado & Davhana-Maselesele 2016). The estimated overall HIV prevalence rate has increased to 12.6% and the number of PLWH was projected to be 7.06 million in 2017 (StatsSA 2017). The epidemic of HIV and AIDS has posed a threat to the whole healthcare system of South Africa. Nurses are burdened with HIV and AIDS caregiving role; they experience increased workload, shortage of staff and equipment, perceived stigmatisation and fear of contagion and emotional exhaustion and fatigue. When nurses are stressed, they display a variety of symptoms that impact the organisational efficiency and effectiveness of caregiving (Ramathuba & Davhana-Maselesele 2013a).

Valjee and Van Dyk (2014) indicated that HIV and AIDS care brought about a significant and added burden to individuals caring for PLWH. Furthermore, healthcare workers are susceptible to issues such as caregiver burden, work-related stress and burnout, anxiety, depression and reduced psychological functioning, which results in psychosomatic distress and a decline in their capability to provide the required care. Staff shortages in public hospitals of South Africa are a great concern, leaving a limited number to face an increasing burden of care (Makhado & Davhana-Maselesele 2016; Ndou 2017). Koto and Maharaj (2016) also reported that approximately 60% of registered nurses (professional nurses) posts are unoccupied and, on the other hand, within the healthcare system there are fewer than 100 (0.5% physicians per 10 000 population) physicians employed in the country. The situation is similar in the Limpopo province where health professionals migrate to other countries and the private sector, leaving the poor under-resourced public hospitals.

Furthermore, the HIV pandemic has negatively affected healthcare workers as they are faced with additional work-related risks such as accidental exposure to blood or blood products or handling non-sterile injecting equipment, as there are shortages of protective clothing such as gloves, sometimes they are of low quality and tear off when wearing (Koto & Maharaj 2016; Makhado & Davhana-Maselesele 2016; Ramathuba 2011; Rasalanavho 2016). Therefore, the risk of contagion is high coupled with the need for skilled health workers who need frequent updates on issues relating to HIV and AIDS to provide care comprehensively and effectively. However, research has shown that nurses lack the necessary knowledge and skills required for caring PLWH (Makhado & Davhana-Maselesele 2016; Mavhandu-Mudzusi, Netshandama & Davhana-Maselesele 2007; Ramathuba & Maselesele 2013b) and that knowledge will alleviate anxiety and stress related to contagion. Furthermore, nurses become emotionally drained when not provided with resources to render nursing care, lack of resources compromise nursing care and eventually lead to burnout and attrition. Emotional exhaustion coupled with poor working conditions and poor managerial support contributes to employee burnout. Employees present with behavioural characteristics such as absenteeism, sickness absenteeism and cynicism and do not show commitment and dedication to their work. Job satisfaction is always related to a supportive organisational climate. Zagenczyk et al. (2010) concurred that when healthcare professionals notice that they are being supported, they tend to be dedicated and committed to the organisation and identify with it whilst helping their organisation to succeed. Sluss, Klimchak and Holmes (2008) cited in Ramathuba (2011) concurred with the notion that personnel see high organisational support when they are provided with the appropriate resources, which can be through rewards, progressive opportunities and valued contribution and being shown by the organisation that it cares about their holistic well-being through positive resource allocation. Thus, support increases the employee’s feelings of responsibility and positive mutuality.

## Research problem

Human immunodeficiency virus and AIDS caregiving has become the basis of stress and burnout amongst nurses as they are burdened by an increased workload of HIV and AIDS care (Makhado & Davhana-Maselesele 2016; Valjee & Van Dyk 2014). Presently the South African government has scaled up the provision of antiretroviral therapy (ART) and initiation of PLWH on ART, but there is limited capacity to carry the task required and the number of patients defaulting treatment is high and these patients present at the health facilities in very critical conditions. The researcher has observed increased turnover and absenteeism in the medical wards for two years. Nurses reported ill almost every week, two or more nursing staff members reported ill and other nurses opted to relocate to the primary healthcare clinics in the district and leaving the hospital setting. Medical wards are very heavy and strenuous; the work demand is more because different chronic conditions demand skills and knowledge specifics of the diseases as treatment and management protocols are continuously changing and providing comprehensive care. These demands place nurses at the risk of accidents and medico-legal hazards. Nurses who experience more job demand and less job control are more likely to experience greater levels of stress than workers with fewer demands and more job control. Nurses who are in lower categories were more likely to be absent from work because of job stress as they are the ones doing most of the manual work other than their senior counterparts in supervisory or management positions. The observations are overtly related to increased workload and fatigue, emotional and psychological burden and witnessing death and dying almost every day and lack of supervisory support. Coping within the healthcare working environment is tough for nurses due to the lack of organisational support, such as stress management, in-depth knowledge about HIV and AIDS, given its fast evolvement and with just a few nurses trained and empowered to deal with the identified problem. Taking into cognisance the impact that HIV has made on healthcare delivery in this country, it is important to develop a support model which will assist nurses to provide quality healthcare services to PLWH.

## Aim

This study aimed to develop a support model for nurses caring for PLWH at a regional hospital in Vhembe district of the Limpopo province, South Africa.

## Research method

The study employed concept analysis and the identified concept of interest within the caring context of HIV and AIDS was conceptualised using the six elements of practice-oriented theory as described by Dickoff, James and Wiedenbach (1968), namely, the context, agent, recipient, dynamic, procedure and the purpose (Ramathuba 2011). The article is based on the parent study that explored and described the experiences of nurses caring for PLWH that was conducted at a regional public hospital in Vhembe district, which is comprised of five district hospitals transferring patients to the hospital for further management. The hospital is a referral hospital in the region (Ramathuba & Davhana-Maselesele 2011). A qualitative approach, exploratory, descriptive design and contextual design were used amongst 15 participants who were purposively selected from a regional hospital in the Vhembe district (Ramathuba & Davhana-Maselesele 2011). Participants included nine professional nurses, four enrolled nurses, two enrolled nursing auxiliaries, and they all had more than 10 years of experience in caring for PLWH (Ramathuba & Davhana-Maselesele 2011). In-depth individual face-to-face interviews and audio-recorded and field notes were used to collect the data. This was followed by the identification of the central concepts that emanated from the qualitative findings, which were conceptualised using Rodgers and Knafl (1993) and relationships were described between the concepts and thereafter the concepts were classified and described within the six elements of practice theory as described by Dickoff et al. (1968), which include context, agents, recipients, dynamics, procedure and outcome in Phase 3.

### Concept analysis

Concept analysis was performed by exploring nurses’ experience of caring for PLWH in a regional hospital in the Vhembe district. This was carried out in two stages, namely, identification and definition of the central concept and the classification of concepts (Rodgers & Knafl 1993). The findings that emanated from phase 1 (qualitative study) revealed that the burden of caring for PLWH has put much strain on the physical, emotional and psychosocial well-being of nurses coupled with lack of managerial support, which has resulted in nurses having low morale and burnout that leads to increased absenteeism. The concept ‘support’ was identified as the central concept and defined as a network of mutual exchange that is based on honesty, rewards and policies that value the contributions and cares about the well-being of its employees.

### Model development

The concepts were classified according to the six survey list elements proposed by Dickoff et al. (1968). The elements entailed the agents, recipients, process, dynamics, context and terminus. Thus, the findings from the qualitative study were conceptualised within the six elements of practice-oriented theory (Dickoff et al. 1968). As indicated in Table 1, the concepts were derived from the qualitative study (Ramathuba & Davhana-Maselesele 2011), which reported the experiences of lack of resources in caring for PLWH; experiences of emotional exhaustion and fear of contagion; experiences of physical and psychosocial distress, self-stigmatisation, isolation and dissociation; and the experiences of lack of educational support and empowerment, concept analysis of ‘support’ and other existing literature that supported these findings (Koto & Maharaj 2016; Makhado & Davhana-Maselesele 2016; Mavhandu-Mudzusi et al. 2007; Ramathuba 2011; Ramathuba & Maselesele 2013b; Rasalanavho 2016; Valjee & Van Dyk 2014).

**TABLE 1:** Conceptual framework to support nurses caring for people living with HIV.

Model guiding questions	Model indicators
In what context is the activity performed?	*Context*: HIV and AIDS care in healthcare institutions (organisation)
Who performs the activity?	*Agent*: Nursing service managers
Who is the recipient of the activity?	*Recipient*: Nurses
What is the energy source of the activity?	*Dynamic*: Power imbalance, leadership style and communication and listening skills from nurse managers
What is the guiding procedure?	*Process*: Phases in the support process: (1) initiation, (2) execution and (3) sustenance
What is the endpoint of the activity?	*Purpose*: A supportive and non-threatening work environment that promotes access to HIV and AIDS education, counselling, treatment and care and committed nurses.

*HIV, human immunodeficiency virus; AIDS, acquired immunodeficiency syndrome.*

*Source*: Ramathuba, D.U., 2011, ‘A model to support nurses caring for people living with HIV/ AIDS in Vhembe district, Limpopo Province’, PhD thesis, Unpublished, North-West University.

### Ethical considerations

Ethical approval to conduct the study was obtained from North-West University (ethical clearance number: NWU-00039-10-S9) and the Limpopo Provincial Department of Health. Permission to collect data was sought from the regional hospital. Informed and written consent was sought from the participants after explaining the purpose and having read the information leaflet of the study. Anonymity and confidentiality were assured to the participants that their names will not appear on the transcript, that they will be identified by codes and numbers and that the name of the participated institution will not appear anywhere.

### Description of a support model for nurses

#### Structure of the model

The description of the structure of the model is based on the theoretical definitions, relationship statements, purpose of the model, assumptions of the model and the process description.

#### The purpose of the model

The purpose of the model is to provide a frame of reference that can be used in healthcare services to initiate and develop a supportive working environment through mutual goal setting in a conducive climate.

#### Assumption of the model

The researchers made assumptions regarding the support between managers and nurses in health institutions to promote a supportive workplace for nurses to be able to cope with caring for PLWH:

- The nurses and managers have their own beliefs and values about the support process in the organisation.
- The organisational climate and attitudes of both managers and nurses can influence the support process.
- Nurses as carers need belonging and recognition, a need to be supported.
- High-quality manager–nurse social exchange is assumed to provide an appropriate basis for the initiation of the support process.
- The support process creates new meaning and understanding amongst participants towards caring for PLWH.

### Relational statement

Relational statements provided links amongst and between the concepts within a model. The following relation statements were formulated for the support model for nurses to cope with caring for PLWH:

- Support process can be initiated when participants understand the need for belonging and identity (perception of oneness with the organisation).
- Dynamics drives the exchange process between nurses and managers.
- Psychosocial support enhances a sense of professional identity and competence.
- Organisational support increases one’s feelings of self-worth and self-esteem.
- Perceived organisational support is related to job satisfaction.
- Job satisfaction is related to organisational commitment.
- Supportive organisational climate is inversely related to organisational cynicism.

### Description of the model

#### Context

Context refers to where the activity is being performed and in this case it is the HIV and AIDS care in healthcare institutions (organisation). The organisational culture influences the nature and outcomes of performances and can also influence the provision or influence of support interventions. The context is constituted by the nursing cadres’ cultural context, values, legislation and policies in the work environment (organisational culture) and the era of HIV and AIDS, which is coloured yellow. The health services’ vision, mission, strategy, goals and values give guidelines to norms and behaviour including social responsibility to the community and well-being of employees. The health service context operates within the legal framework for support to take place (blue colour). The empirical findings are as follows: Nurses were not satisfied with how management was dealing with issues of HIV and AIDS, there was much stigmatisation and dehumanisation to those nurses caring for PLWH and there was a lack of shared value and commitment to HIV and AIDS care.

The provision of support should not only take place within the legal and professional sphere. Support should be seen to promote human dignity, equality and respect of human right and labour laws. All these require organisations or institutions to provide nurses as caregivers a safe working environment that supports and fosters professional and ethical values by maintaining a climate of mutual trust, respect, sharing ideas and assertiveness towards HIV and AIDS issues. Castro and Martins (2010) indicated that organisational climate is the global impressions of the organisation that members form through interacting with each other and organisational policies, structures and processes; it refers to the feeling of an organisation and influences individual behaviours. Berberoglu (2018) also indicated that organisational climate can be considered an aspect of culture and defined as team spirit but at the organisational level, and one of the most important aspects in an organisation is the shared beliefs and values within the organisation.

The current health situation, HIV and AIDS pandemic is impacting the organisation and influences performance and sense of well-being of nurses. Findings indicated that nurses were physically, psychologically and emotionally burdened (Ramathuba & Davhana-Maselesele 2011). Organisational climate has a significant impact on the well-being of employees that in turn has a direct influence on the quality and quantity of work performed in the organisation (Berberoglu 2018). Therefore, healthcare settings must take cognisance of the employee’s perceptions of the work situation including the characteristics of the organisation to create an organisational climate that has a direct relationship with the employees. Several studies showed that employees who are supported by their organisation are satisfied with their job and display positive behaviour and commitment to organisational norms (Ayuningtias et al. 2019; Günay 2017).

#### Agent

Agent refers to the person who performs an activity and in this case it is the nursing service managers who are responsible for providing support to carers. The findings revealed that managers were not supportive, even senior members were found gossiping about staff members who were living with HIV and AIDS (Ramathuba & Davhana-Maselesele 2011). Furthermore, there were no debriefing sessions for nurses and nurses complained of staying in medical units for prolonged periods without rotation. Managers must ensure that the rights of individuals are protected and acknowledge the seriousness and the implication of HIV and AIDS. Furthermore, they should provide a supportive work environment for employees living with HIV and AIDS, design policies with other stakeholders and employees to provide input on issues of when an employee is said to be too sick to work. Shumba, Kielmann and Witter (2017) exposed that professional and social divisions were found to be the source of conflict between health workers and facility management. These conflicts were characterised by undesirable reactions articulated towards governance and management.

Nursing managers in a leadership position must possess a certain characteristic that will create a supportive environment. They need to be responsible for creating and changing the organisational culture that is positive through the creation of a robust vision for the forthcoming HIV and AIDS management support programmes and interventions.

#### Recipient

Nurses are the recipients who care for patients within the context of HIV and AIDS. The agent and recipient should develop a sense of team spirit and be committed to the realisation of a supportive environment. The findings revealed that self-awareness, mutual trust, respect and good interpersonal relationships are the key features to the initiation and development of this support process (Ramathuba & Davhana-Maselesele 2011). Participants indicated the need to be respected as individuals and management must refrain from pointing fingers without engaging them. Lack of trust in the management resulted in poor interpersonal relations (Ramathuba & Davhana-Maselesele 2011). Nkomazana, Mash and Phaladze (2015) believed that workers’ attributes and the need for transformed organisations, transformed leadership from a bureaucratic controlling style to a more supportive type restores employee trust. Furthermore, this transformation should emphasise worker’s experience with importance on accountability, transparency, professional development, staff recognition and shared decision-making (Nkomazana et al. 2015). The arrows of agents and recipients facing each other indicate the influence both parties can exert to ensure that the process of initiation and implementation of support in the era of HIV and AIDS is conducted in health services.

#### Dynamics

Dynamics are driving powers behind the activity that can be chemical, physical, biological and psychological for any person or thing operating as an agent or part of the framework in realising the goal (Dickoff et al. 1968). The dynamics are depicted as side arrows directing the process (procedure) of support. The agent facilitates the support process by forming an environment that is conducive through a dynamic process of provision of the needed support. The dynamics are inherent in the process of providing support to nurses that provide care to PLWH, and the findings revealed power imbalance, leadership style and trust. Participants indicated that there should be open communication between managers and staff; managers should listen to their concerns, acknowledge their contributions unlike saying they will earn their benefits from heaven. Nurses felt to be lacking trust and confidence in management. Shumba et al.’s (2017) study revealed that respondents felt little communication, and if it happened it occurred poorly. Support interventions ought to be characterised by a trusting relationship, reduced power imbalances for nursing personnel not to feel threatened by organisational protocols, to develop climate for teamwork with common values and a vision of providing active support to nurses living with and caring for PLWH. The truthfulness and transparency of the communication with staff are critical, such as availability, consistency, discreetness, fairness, integrity, loyalty and openness. Trust is promoted through a social exchange process where both the employee and the organisation interpret the behaviour of the other to determine whether reciprocation is necessary. Nkomazana et al. (2015) indicates that increased supportive and innovative culture combined with considerate supportive supervisors or managers have always been associated with amplified job satisfaction and organisational commitment. Organisational culture and leadership styles determine the work environment; this might be helpful or harmful to organisational effectiveness and job satisfaction.

#### Procedure

The procedure is to accentuate the pathway, steps or pattern that the activity is performed (Dickoff et al. 1968). The procedure is depicted as the central arrow pointing upwards with the steps in the process. The activities that were viewed as important by the participants in developing a model for supporting nurses caring for PLWH were identified in the first phase as self-awareness, mutual goal setting, interactive communication and facilitation skills, role modelling and role identification (Ramathuba 2011).

Effective communication is essential during the development or initiation of a support strategy as nurse managers and nursing personnel can be able to share and exchange pertinent information relating to their concerns and aspirations. Phase 2 is the execution and implementation of the support process that requires teamwork and participative management to mobilise the objectives of the supportive process (Ramathuba 2011). Mutual goal setting makes employees and management to be goal-orientated and it improves employee’s satisfaction and morale. Cooperative planning and joint decision-making are necessary for the formulation of a strategic plan for accomplishing the goals of the support strategy, mapping the process and members are allocated roles and responsibilities. Nkomazana et al. (2015) reported that participants in their study desired organisational transformation, which focuses on constructing a sense of shared purpose, internal community, shared decision-making and participation of employee rather than a limiting culture. Belrhiti et al. (2020) suggested that supervisors and managers should empower their subordinates instead of directing or managing them through the act of commanding and controlling; they should inspire the spirit of sharing of information, providing effective support to junior staff and maintain disseminated leadership. Furthermore, transformational managers, supervisors and leaders who display individual consideration and undoubtedly communicate their vision have the potential to intensify the self-esteem of staff and perceived supervisor support is related to satisfaction and self-sufficiency needs. This may contribute to staff commitment, mutual trust and extra-role performance (Belrhiti et al. 2020). Phase 3 indicates sustenance of the support process through requiring feedback and reflections from recipients on a continuous basis through surveys to improve as well as to monitor and evaluate policies and procedures in the provision of support.

## Expected outcome

**FIGURE 1:**
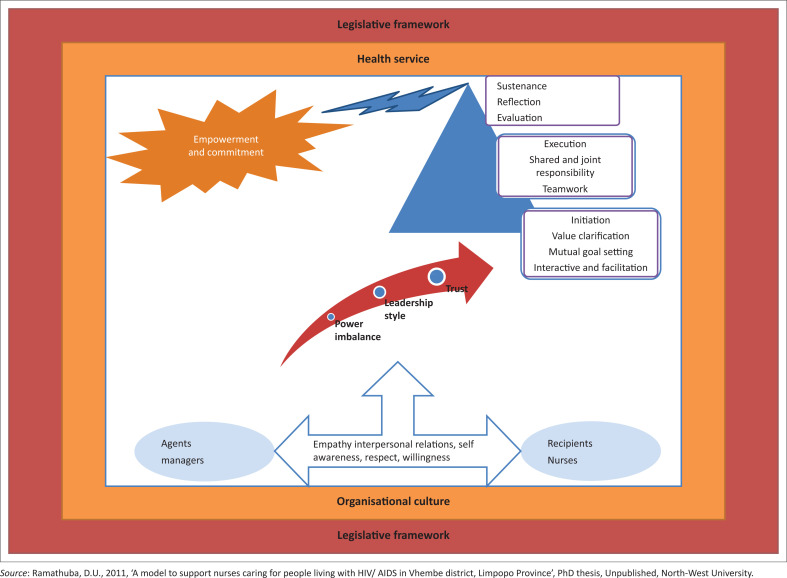
Schematic representation of the model to support nurses caring for people living with HIV and AIDS.

The model intends to demonstrate a supportive and non-discriminatory work environment that promotes job satisfaction, commitment and empowered nurses. Managers should empower and support nursing personnel with resources and skills. Empowerment enhances knowledge, skills and attitudes towards a supportive work environment that is based on open communication, honesty, participation and commitment. The outcome is depicted by Figure 1. Beheshtifar and Herat (2013) concurred that personnel who see the organisation as a caring organisation for their well-being are presumed to be more likely to respond in numerous forms of pro-social behaviour directed towards the organisation, through developing a robust sense of commitment towards the organisation.

### Model evaluation

The evaluation of the model described in Chinn and Kramer (2011) was used to reflect generality, importance, simplicity and clarity.

### General

The scope of the concepts and the purpose within the model show that the model has a broad scope of application. The model can be applied in other cooperate organisations or institutions as HIV and AIDS are affecting individuals, families and business organisations and workers are part of this broader community.

### Importance

The model would assist in providing support to nurses to ease the pain of caring and promote a supportive work climate that is conducive to their physical and mental well-being.

### Simplicity

Model simplicity implies that the number of elements within each descriptive category, the concepts and their inter-relationships were kept to a minimum and the arrows were showing the direction of activities.

### Clarity

Structural clarity was achieved by the concept map that was derived from concept analysis, and it provided meaning on how the provision of support to nurses caring for PLWH in health services can be achieved for them to be able to cope. Minimal concepts were used to enhance clarity.

## Limitations

The study was conducted at a regional hospital in one province; moreover, the sample size was quite small because the participants were selected based on convenience sampling, to which extent the study findings have limited generalisability.

## Recommendations

### Nursing practice

Providing support to nurses in the context of HIV and AIDS requires legislating the provision of work-based programmes that ensure that employers provide a baseline level of support and thus exterminating discrimination, stigmatisation and providing required practical support by availing needed resources towards caring for PLWH. Workshops targeted at enabling the expression of feelings, conflict resolutions and a positive reassessment may promote both stress response modification and stress coping.

### Nursing education

The model has the potential to provide an appropriate understanding of how work-associated stress affects nurses caring for PLWH, and what factors in their work environment cause or contribute to the greatest burden to gain more knowledge about nurses’ working conditions, occupational stress and job satisfaction. This knowledge might be used to decrease occupational stress and increase job satisfaction amongst nurses caring for PLWH. Strategies to equip nurses with recent and up-to-date information through seminars, workshops, in-service training and short courses may be beneficial.

### Nursing research

The findings indicated that nurses lacked managerial support as they experience shortages of resources , physical and emotional burden and lack educational support that resulted in job stress or the burden of caring. However, there is no existing evidence that empirically illustrates how managerial stress affects staff stress. Given the current emphasis on improving the work environment, there is a dire need to explore the aspects of the nurse managers about stress and burnout.

## Conclusion

Findings of this study indicate that the burden of HIV and AIDS care related to high job demand, insufficient job control, and inadequate social and educational support are the key factors associated with an increased risk of poor job satisfaction and morale. Also, high job demands and poor organisational support were related to increased absence because of illnesses of nurses. A lack of reward and appreciation by supervisors increased the emotional stress and resulted in a lack of commitment associated with increased absence from duty because of illnesses amongst nurses. Poor organisational support deteriorates the quality of nurses’ lives on a personal level and imposes a direct economic cost on the employer by decreasing overall nursing workforce productivity. Employees who experienced low levels of organisational support at work are more vulnerable to poor psychological and physical health problems, such as heart disease, migraines, hypertension, irritable bowel syndrome, muscle, back and joint pain, and duodenal ulcer, which results in a higher risk of sickness absenteeism.

The study findings could be useful for guiding intervention programmes related to the quality of the nursing workforce’ lives, with the management of absenteeism and addressing unfavourable work-related psychosocial job stress.
